# Lung ultrasound in outpatient approach to children with suspected COVID 19

**DOI:** 10.1186/s13052-020-00938-w

**Published:** 2020-11-23

**Authors:** Giuseppe Gregori, Roberto Sacchetti

**Affiliations:** Local Health Unit, Department of Primary Care, Medicina di Gruppo Pediatrica Piccolo Daino, Via Conciliazione 45/A, 29121 Piacenza, Italy

## Abstract

**Background:**

Children with COVID 19 infection (CV19) generally have a mild disease whose main symptoms are fever and cough. Dyspnoea and hypoxemia are rarely reported and few data are available on the frequency and extent of lung involvement in children with CV19. In addition, due to the limited availability of diagnostic tests in Italy during the pandemic period and the relative reliability of the test results, the diagnostic suspicion of CV19 infection in most of the children was difficult to confirm. The aim of this study is to evaluate if lung ultrasound (LUS) was able to highlight typical interstitial lung lesions in children with persistent cough and suspected CV19, providing corroborating evidence of CV19 infection.

**Methods:**

We retrospectively analysed the data of 32 children who came consecutively to our outpatient observation in the period between March 1st and April 30th, 2020 because of the presence of persistent cough for at least 3 days and with suspected CV19. All the children undergone clinical examination, oximetry measurement and LUS.

**Results:**

Twenty over thirty-two children had US lesions compatible with the presence of CV19, many of them without clinical signs of respiratory distress. LUS is much more sensitive than clinical examination to detect lung injury in children with suspected CV19.

**Conclusion:**

In the absence of reliable, highly sensitive diagnostic tests or when nasal swab is unworkable or there are too many requests to be performed quickly due to the pandemic, LUS should be used in children with persistent cough for whom a CV19 is suspected because it can highlight undiagnosed interstitial lung lesions and reinforce the diagnostic suspicion of CV19 This approach can be very useful in outpatient settings and in areas with limited medical resources such as developing countries.

## Background

In Italy, the alert status for the presence of COVID19 (CV19) was officially declared on January 31, 2020 (https://www.gazzettaufficiale.it/eli/id/2020/02/01/20A00737/sg). On February 23, 2020, at the same time with the creation of the first “red zone” in Italy (Codogno), schools were also closed in the Piacenza district, which borders the “red zone” (https://www.normattiva.it/uri-res/N2Ls?urn:nir:stato:decreto.legge:2020-02-23;06!vig=). The country began a full lockdown on March 8, 2020 (https://www.gazzettaufficiale.it/eli/id/2020/03/09/20G00030/sg). Piacenza district recorded one of the highest cumulative mortality rates (> 3 deaths per thousand inhabitants) in Italy (https://www.istat.it/it/archivio/242149). The number and severity of cases occurred simultaneously in this area among adults and elderly people had a significant impact on the regional health services for several weeks. In this emergency situation, most children with suspected CV19 were not tested due to the lack of nasal swabs and mild symptoms, which rarely required hospitalization. Fever and cough are the prevalent symptoms in children with CV19 [1, 2] but gastrointestinal and skin symptoms are also often described, compared to those observed in adult patients [2]. Respiratory complications such as dyspnoea and hypoxemia are rarely reported and few data are available on the frequency and extent of lung involvement in the course of CV19 in children. The average incubation time for CV19 is a few days [3, 4] and most paediatric cases originate within a family cluster [1]. At the beginning of the epidemic, paediatricians* tried to remotely manage most children with symptoms suggesting a possible CV19 infection. Subsequently, as adequate protective devices started to become available and with safety measures in place, part of the symptomatic and suspect CV19 children were assessed at paediatricians’ surgeries. The use of Lung Ultrasound (LUS) in children is useful for the diagnosis of the main and most frequent infectious lung diseases, in pleural effusions, and in pneumothorax [5, 6]. The interstitial lung pathology that characterizes CV19 on CT examination, can determine peculiar imaging even at US level (thickening of the pleural line, frequent or coalescent B-lines especially at the bases posteriorly, white lung, sometimes areas of consolidation) [7]. The aim of our study is to retrospectively analyse the anamnestic, clinical, epidemiological data and the images detected on LUS of children who presented cough for at least 3 days and suspected CV19, who were managed as outpatient in our medical office in the Piacenza district from March 1st to April 30th, 2020. In Italy the NHS provides for the presence of paediatricians for the population from 0 to 16 years old who perform the same task as general practitioners for adults. They can carry out their activity individually or in groups / associations.

## Materials and methods

The data of 32 children living in Piacenza district who consecutively came to our outpatient observation between March 1st and April 30th, 2020 were examined retrospectively due to the presence of cough for at least 3 days and epidemiological criteria for suspected CV19 shared by international health authorities and adopted by the Italian Ministry of Health [1, 8, 9]. During the same period, about 70 children aged between 7 months and 14 years of 2200 patients in our catchment area, were reported as suspicious CV19, as well as 2 asymptomatic new born from positive CV19 mothers. The clinical data, including oximetry (to detecte any signs of hypoxyemia) and LUS outcome were collected. A 53 mm ultrasound linear probe (5–10 Mhz) was used for LUS. US images were divided into 4 patterns based on the progressive loss of aeration of the lung [10]:

- Pattern A: normal
- Pattern B: presence of multiple B lines with or without thickening of the pleural line
- Pattern C: coalescent B lines (white lung)
- Pattern D: presence of areas of consolidation > 1 cm

After the outpatient visit, all children were reported to the Public Hygiene Service to undergo a nasal swab for CV19, but it was possible to carry out a diagnostic swab only for 10/32 children, 5–7 days after LUS. The other 22 children underwent a nasal swab only after quarantine period, as the Service had other priorities aimed at adults and the elderly.

## Results

**Fig. 1:**
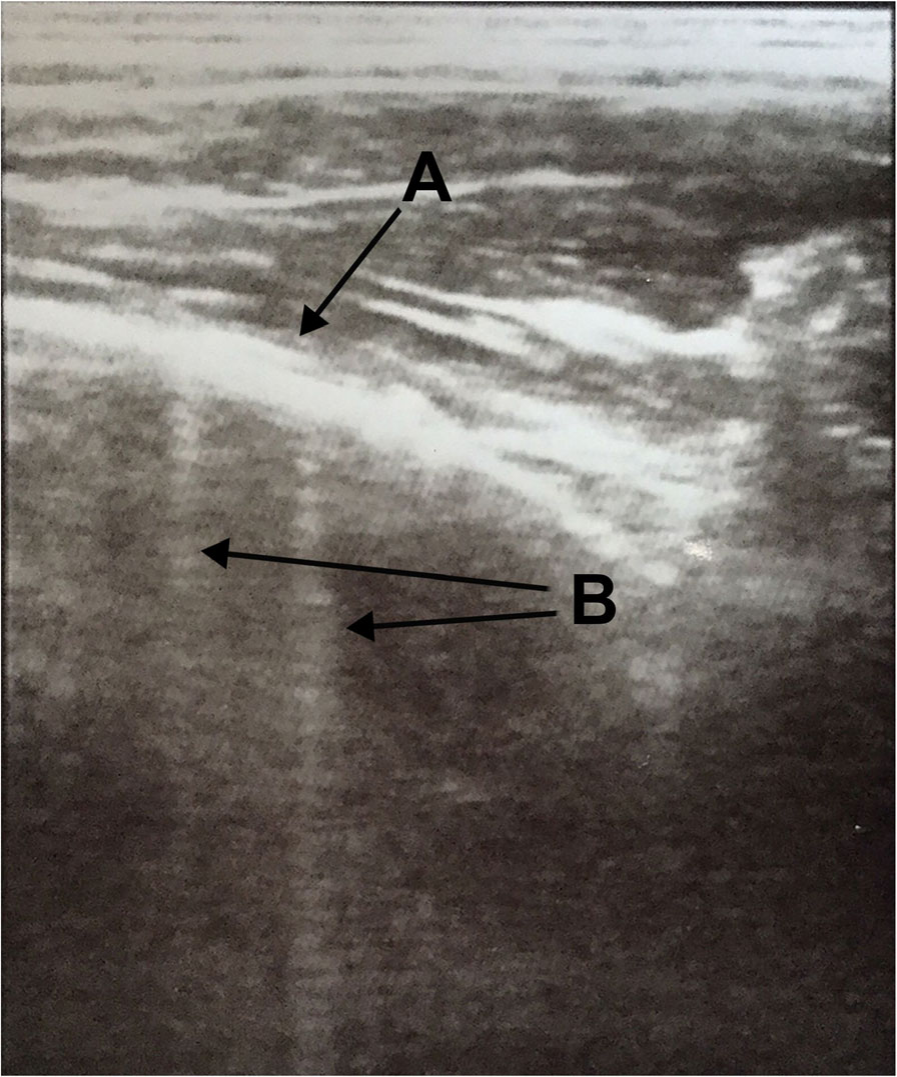
Thickening of the pleural line (**a**) and B-lines (**b**)

**Fig. 2:**
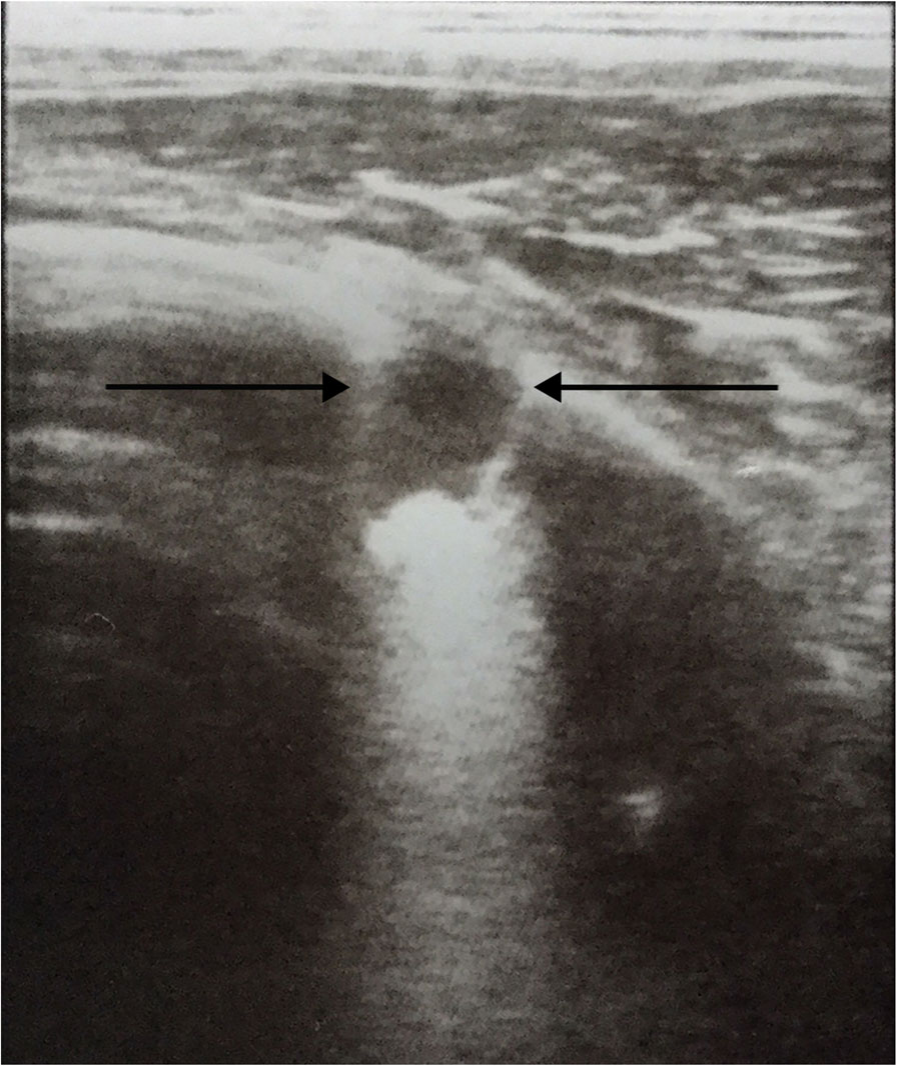
Small subpleural consolidated area

**Fig. 3:**
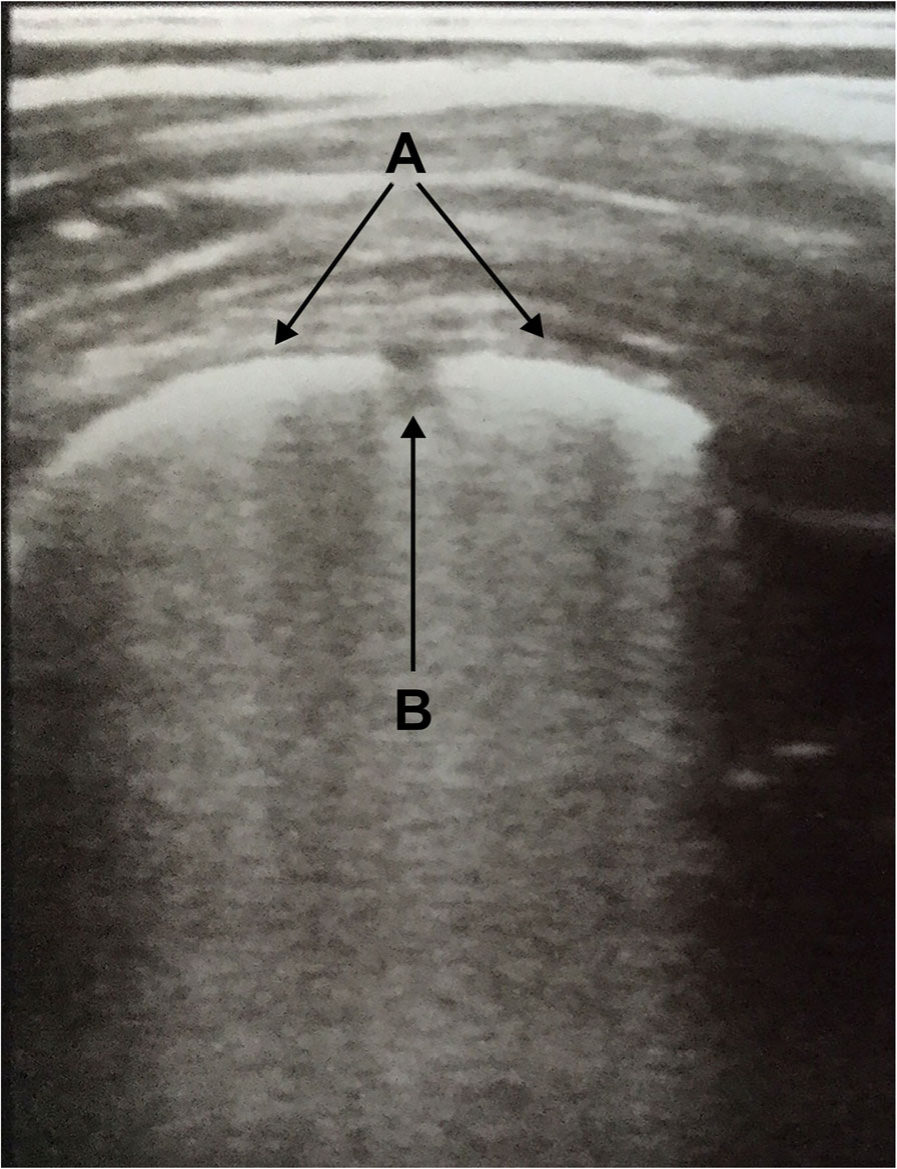
Thickening of the pleural line (**a**), broken in the middle (**b**). Below coalescent B-lines (white lung)

Among the 32 children examined, 23 were male and 9 were female (age range of 6 months-14 years 8 months) and all presented epidemiological criteria for suspected CV19 and cough for at least 3 days. Twelve cases presented fever > 37.5 °C, nine cases presented headache, four cases had pharyngodynia, four cases had asthenia, two case had chest pain and one case had respectively erythema pernio-like (dermatological lesions of the feet and in lesser measure of the hands similar to frostbite), diarrhoea, conjunctivitis. Eight children presented oximetry < 97% (minimum observed value was 95%). LUS was normal (pattern A) in 12 children. In 16 cases, LUS showed a Pattern B (Fig. 1) one of which with associated multiple small subpleural foci (Fig. 2), 1 case presented a Pattern C (white lung) (Fig. 3) and only 3 cases had a Pattern D. In 2 children, the areas of consolidation were bilateral, in 1 case consolidation was mono lateral. In two children with pattern D the suspicion of pneumonia had initially arisen at auscultation: after LUS also the chest X-ray was performed confirming the presence of pneumonia. Among the 20 children with positive LUS (13 M and 7 F, age range: 6 months − 10 to, 1/2), nine also presented fever > 37.5C°. No significant relationship was observed between the degree of pulmonary commitment assessed at the US level and the clinical symptoms of the patient..CV19 was detected in 2/10 nasal swabs.

## Discussion

It should be considered that the timing of the swab is not that of the LUS. The swab is very important in the early stages of the disease while the LUS in our experience was used several days after the start of the infection: therefore LUS cannot and doesn’t want to replace the execution of the nasopharyngeal swab in the diagnosis of CV19. Unfortunately the great number of suspected cases of CV19 simultaneously emerged at the beginning of the pandemic period in our district, many of which were elderly and with critically health conditions, overshadowed the generally less demanding paediatric CV19 pathology. As opposed to adults and the elderly, who often have interstitial pneumonia, children with suspected CV19 rarely present respiratory complications, such as dyspnoea and hypoxemia. Consequently, as not enough swabs were available for all suspected CV19 cases, only in 10/32 children a diagnostic swab was done, in any case several days after LUS evaluation and often with a negative result. All patients had had direct or indirect contact with CV19 adult subjects(mainly parents, grandparents, uncles) and had not attended kindergartens or schools for at least 10 days,most of them for at least 3–4 weeks. The literature about nasal/oropharyngeal swab during CV19 infection states that a negative test doesn’t exclude the disease [11]. Furthermore it seem important to carry out nasal swab at the beginning of the disease when the viral load is higher [12]. In any case,when a diagnostic test isn’t quickly available, early recognition and isolation of cases with suspected CV19 can be very important to reduce the spread of the infection and the clinical management of the patient.

In our experience, LUS performed on patients with strong epidemiological and clinical suspicion for CV19 and experiencing cough for at least 3 days has detected a CV19-compatible pattern in over half of the children examined. Two children with pattern D after LUS underwent also chest X-ray confirming the presence of pneumonia: this choice was made to exclude increased lung involvement as the clinical conditions were precarious. In some instances, LUS results were unexpected: subjects with mild / medium respiratory symptoms and positive epidemiological data presented a greater lung involvement. This experience has been confirmed in adult patients by other authors [13]. We chose LUS classification of Bouhemad B et al. [10] because it seems simple and reflects some educational videos created by Italian university colleagues and spread via the internet especially on You Tube. We did not have time to adjust our ultrasound assessment to the proposal of Soldati G et al. for “International Standardization of the Use of Lung Ultrasound for Patients With COVID-19 guidelines” published during the pandemic [14].. LUS allowed a faster diagnostic procedure and avoided the use of more invasive methods (Rx and CT).

LUS in childhood also offers other advantages: it yields real-time and dynamic images, allows easy repetition, especially in the case of monitoring, can be performed even with a poorly collaborative child, has a favourable cost / benefit ratio, and does not require exposure to radiation nor sedation. LUS has also been essential in the safe management of the CV19 outbreaks [15] and should become a first-line method in the assessment of respiratory infections in not hospitalized children. *For epidemiological purpose decided by National Health Service,1 month after the end of this retrospective analysis 19/32 children underwent to sierological test of CV19 antibodies (CMIA Abbott): 9/19 had significant IgG levels anti-CV19,6 of which belonging to the group of children who had had a pathological LUS*.

## Conclusion

In the absence of reliable, highly sensitive diagnostic tests or when there are too many requests for nasal swab to be performed quickly due to the pandemic, the LUS point of care seems very useful and should be recommended in children with suspected CV19 and persistent cough. LUS may highlight undiagnosed interstitial lung lesions and reinforce the diagnostic suspicion of CV19. Its favourable cost / benefit ratio, the fast execution time and the rapid interpretability of the images make this approach very advantageous in the outpatient setting and in areas with poor medical equipment, such as developing countries.

## Data Availability

The datasets used and/or analysed during the current study are available from the corresponding author on reasonable request.

## Abbreviations

CV19: Covid19
LUS: Lung Ultrasound
